# Ethnic differences in overweight and obesity and the influence of acculturation on immigrant bodyweight: evidence from a national sample of Australian adults

**DOI:** 10.1186/s12889-016-3608-6

**Published:** 2016-09-05

**Authors:** Karen Menigoz, Andrea Nathan, Gavin Turrell

**Affiliations:** 1 School of Public Health and Social Work, Queensland University of Technology (QUT), Victoria Park Road, Kelvin Grove, QLD 4059 Australia; 2 Institute for Health & Ageing, Australian Catholic University (ACU), Level 6, 215 Spring Street, Melbourne, VIC 3000 Australia

**Keywords:** Obesity, BMI, Bodyweight, Ethnicity, Immigrant, Minority, Acculturation, Prevention, Inequality, Australia

## Abstract

**Background:**

Despite growing international migration and documented ethnic differences in overweight and obesity in developed countries, no research has described the epidemiology of immigrant overweight and obesity at a national level in Australia, a country where immigrants comprise 28.1 % of the population. The aim of this study was to examine ethnic differences in body mass index (BMI) and overweight/obesity in Australia and the influence of acculturation on bodyweight among Australian immigrants.

**Methods:**

Data from the national Household Income and Labour Dynamics in Australia (HILDA) survey were used to examine mean BMI and odds of overweight/obesity comparing immigrants (*n* = 2 997) with Australian born (*n* = 13 047). Among immigrants, acculturation differences were examined by length of residence in Australia and age at migration. Data were modelled in a staged approach using multilevel linear and logistic regression, controlling for demographic and socioeconomic variables.

**Results:**

Relative to Australian born, men from North Africa/Middle East and Oceania regions had significantly higher BMIs, and men from North West Europe, North East Asia and Southern and Central Asia had significantly lower BMIs. Among women, the majority of foreign born groups had significantly lower BMIs compared with Australian born. Male and female immigrants living in Australia for 15 years or more had significantly higher BMIs and increased odds of being overweight/obese respectively, compared with immigrants living in Australia for less than 5 years. Male immigrants arriving as adolescents were twice more likely to be overweight/obese and had significantly higher BMIs than immigrants who arrived as adults. Male and female immigrants who arrived as children (≤11 years) had significantly higher odds of adult overweight/obesity and BMIs.

**Conclusions:**

This study provides evidence of ethnic differences in overweight and obesity in Australia with male immigrants from North Africa/Middle East and Oceania regions being particularly vulnerable. In addition, this study suggests that greater acculturation may negatively impact immigrant bodyweight and recently arrived immigrants as well as those who arrive as children or adolescents may benefit from obesity prevention intervention. Public health policy targeted at and tailored to these immigrant cohorts will assist in the multi-pronged approach required to address the obesity epidemic.

## Background

Obesity is a significant global health challenge impacting both developing and developed countries [1]. Worldwide, international migration has increased 41 % from 2000 to 2015, with over 244 million people now living in a country other than where they were born [2]. The prevalence of overweight and obesity is ethnically [3–6] as well as socioeconomically patterned [7–10] and understanding the nature of these relationships is important in designing effective obesity prevention policy.

Ethnicity and bodyweight research is dominated by studies from the United States and Europe which demonstrate stark, multi-generational inequalities in overweight and obesity among some ethnic minority groups [3, 4, 11–15]. Few studies however have focused on the Asia Pacific region and no published studies have defined ethnic differences in bodyweight in a national sample of Australian adults. Australia has high rates of overweight and obesity (70.3 % of men and 56.2 % of women) [16] and a particularly high immigrant population with 28.1 % of the population born overseas [17] (in contrast for example, to the United States which has 12.5 % born overseas [18]). It is somewhat surprising therefore, that epidemiological studies of ethnic difference in bodyweight in Australia have focused largely on children [19–22] and the three known studies that focused on adults [23–25] have a number of substantive and methodological limitations. In particular, previous Australian studies have been based on single State surveys, were not inclusive of all ethnic groups, two studies did not address expected gender differences [23, 24] and two studies were limited to older adults [23, 25].

Alongside ethnic differences in bodyweight, a related body of research has examined the influence of acculturation on immigrant bodyweight. Acculturation is defined as a change in cultural patterns arising from exposure to the host country’s lifestyle, environment and culture [26, 27]. Studies primarily from the United States and United Kingdom have consistently shown that upon arrival, immigrants have lower BMI, overweight and obesity relative to their host-country born counterparts [5, 12, 28–31] however longer residence has been shown to be associated with higher BMI [28, 30, 32, 33] – often attributed to acculturation [32, 33]. Acculturation can be assessed with scale measures (typically measuring language, use of media in the host country, values, lifestyle, attitudes and ethnic social relations and networks), as well as via temporal measures such as length of residence in the host country and age at arrival [32, 34]. While scale measures more sensitively measure social structural and cultural changes, temporal measures are more readily available and commonly used in population immigrant health research [32, 34]. Length of residence is thought to influence immigrant overweight and obesity through behavioural change such as adoption of unhealthy dietary habits [35]; contextual effects, such as ethnic social network [5] and neighbourhood effects [36]; and a range of individual differences - in household income, English proficiency, acculturative stress, experiences of discrimination [37] and education, gender and racial/ethnic group [28]. Age at arrival may influence adult obesity risk due to the different adaptive capabilities of children vs adults [38], English language proficiency [39], wage earning potential [39], and the level of acculturation to behaviours such as physical activity, diet and smoking [40]. While this topic has received some attention in the Australian context [24, 25, 35], there are no known studies to date, which have examined at a national level, whether acculturation and obesity relationships hold true in Australia’s unique immigration history and immigration policy environment.

The aim of this present study therefore, is to present for the first time, national-level findings on the gender-specific ethnic differences in BMI and overweight/obesity in Australian adults and the influence of acculturation on bodyweight among immigrants to Australia.

## Methods

### Study design and sample

This paper uses Wave 11 (2011) data from the Household Income and Labour Dynamics in Australia (HILDA) survey. HILDA is a national, household-based longitudinal survey about life in Australia that includes a range of ethnicity and migration related variables and information on economic, social and demographic characteristics. The HILDA methodology is described in detail elsewhere [41]. Briefly, the scope and coverage of the survey are Australian households (and usual residents) in private dwellings, excluding very remote and sparsely populated areas [41]. The panel in wave 1 of the survey consisted of 7 682 responding households and 19 914 individuals. The sample was topped up in wave 11 with an additional 2 153 responding households. The selection method for the top-up sample was similar to the original sample methodology [42]. The survey research team have examined the issue of cross-sectional representativeness and found that combining the main sample with the top up sample served to improve the quality of the cross-sectional estimates (compared to just using the main sample), particularly for estimates of country of birth and year of arrival [43]. The survey researchers also found that the combined sample resulted in estimates which more closely reflected data benchmarked from the Australian Bureau of Statistics Labour Force Survey [43]. The majority of wave 11 interviews were conducted during the period August to November, 2011. Data were collected using personal interviews with each member of the household aged ≥15 years, followed by a self-completion questionnaire which included questions on lifestyle and health habits. In 2011, 10 440 households were included in the study with 64.9 % of these being fully responding households: this resulted in a sample of 17 612 responding individuals.

### Measures

#### Anthropometric measurements

Two common indicators of population weight, mean BMI and prevalence of overweight/obesity were used in this study. Weight and height were self-reported and BMI was calculated as weight in kilograms divided by the square of height in metres and outliers removed [44]. The dichotomous variable for overweight/obesity (or not) was derived as per WHO cut offs (BMI >25 kg m^−2^) [45]. Overweight/obesity as a combined category is clinically relevant due to the established health consequences of exceeding a body mass index of 25 kg m^−2^ (see Ng et al. [1] for an overview). It is also a policy-relevant categorisation reflecting international obesity reduction targets and indicators [45].

#### Ethnicity

The ethnicity variable used in this study was Country of birth (sometimes referred to as Nativity), categorised into regions using the Standard Australian Classification of Countries (based on geographic proximity and similarities in economic, social and political characteristics) [46].

#### Acculturation

There were two acculturation variables. Length of residence in Australia was calculated by subtracting the year the person first came to Australia to live from the year of the survey; and Age at arrival, calculated by subtracting the year of birth from the year the person first came to Australia to live. Consistent with previous research [33, 40], both variables were transformed into categorical variables for the analysis (see Table 1 for definitions).

**Table 1:** Socio-demographic and bodyweight characteristics of men and women: ethnicity and bodyweight analytic sample (*n* = 13 047)

	Men (*n* = 6 216, 47.6 %) 27.1 (5.0) Mean BMI (SD) 65.2%Owt/Obese			Women (*n* = 6 831, 52.4 %) 26.6 (6.3) Mean BMI (SD) 52.8%Owt/Obese		
	%	Mean BMI (SD)	%Owt/Obese	%	Mean BMI (SD)	%Owt/Obese
Country of birth						
Australian born	76.5	27.1 (4.9)	65.3	77.4	26.8 (6.4)	54.2
Oceania (excluding Australia)	3.3	28.3 (5.5)	71.6	2.6	27.0 (5.7)	58.5
North-West Europe	9.5	26.9 (4.4)	67.3	8.4	26.6 (5.9)	53.1
Southern & Eastern Europe	2.6	27.7 (4.3)	74.7	2.5	27.5 (5.7)	64.5
North Africa & The Middle East	1.0	28.8 (8.5)	68.8	0.7	28.0 (6.2)	62.2
South-East Asia	1.7	26.3 (5.6)	53.3	2.7	24.7 (6.2)	35.3
North-East Asia	1.1	25.2 (5.7)	39.4	1.8	21.5 (3.0)	11.6
Southern & Central Asia	1.9	25.7 (6.1)	51.7	1.6	25.0 (6.4)	42.6
Americas	1.1	28.2 (5.5)	71.4	1.4	25.3 (6.3)	43.3
Sub-Saharan Africa	1.2	26.0 (3.5)	58.9	1.0	24.5 (5.1)	35.7
Age						
18–24 years	13.2	24.8 (4.7)	40.3	12.4	23.7 (5.2)	26.7
25–34 years	14.4	26.4 (4.5)	58.8	13.8	25.4 (6.3)	41.8
35–44 years	16.8	27.6 (5.1)	68.3	17.7	27.2 (6.6)	55.1
45–54 years	20.3	27.8 (4.9)	73.3	19.7	27.2 (6.5)	56.1
55–64 years	16.6	28.3 (5.2)	74.2	17.3	28.0 (6.3)	63.5
65–74 years	11.4	27.5 (4.8)	72.3	11.0	27.8 (6.0)	64.4
> 75 years	7.2	26.2 (4.4)	61.3	8.1	26.1 (5.5)	54.7
Remoteness						
Major City	63.4	26.9 (4.9)	63.3	63.8	26.3 (6.2)	50.0
Inner Regional Australia	24.1	27.3 (5.0)	67.4	24.7	27.1 (6.2)	57.3
Outer Regional Australia	10.9	27.6 (5.3)	69.4	9.9	27.6 (7.0)	58.6
Remote and Very Remote Australia	1.6	28.5 (4.6)	78.6	1.6	27.5 (6.4)	57.8
Highest attained education level						
Bachelor +	23.5	26.7 (4.6)	63.5	26.1	25.6 (5.6)	45.4
Diploma	9.4	27.3 (4.5)	68.0	9.5	26.4 (6.4)	51.2
Certificate (trade/business)	28.6	27.7 (4.9)	71.2	16.2	27.3 (6.5)	57.7
School - Year 12 and below	38.4	26.9 (5.3)	61.0	48.2	27.0 (6.5)	55.4
Occupation						
Managers and professionals	27.7	27.1 (4.3)	68.3	23	26.0 (5.6)	47.1
White Collar	13.6	27.0 (5.2)	60.7	30.8	26.3 (6.3)	50.2
Blue Collar	29.8	27.2 (4.9)	65.1	6.9	26.8 (6.8)	52.1
Unemp/Not in Labour Force	28.8	27.0 (5.4)	64.4	39.3	27.2 (6.6)	58.2
Household Income						
> $130,000 k per annum	19.4	27.0 (4.6)	64.4	17.2	25.5 (5.6)	44.5
$72,800–$129,999	35.6	27.2 (4.8)	67.5	32.9	26.4 (5.9)	50.9
$52,000–$72,799	17.1	26.8 (4.9)	63.2	16.0	27.2 (6.6)	55.8
$41,600–$51,999	8.2	27.2 (5.1)	65.4	8.0	27.3 (7.1)	53.4
$26,000–$41,599	11.9	27.0 (5.6)	63.4	13.4	27.3 (6.5)	58.7
$0–$25,999	7.8	27.0 (5.6)	63.4	12.5	27.0 (6.7)	58.1
Neighbourhood Disadvantage						
Quintile 5 (least disadvantage)	22.0	26.4 (4.0)	61.4	21.2	25.4 (5.6)	44.3
Quintile 4	22.1	26.8 (4.7)	63.8	22.2	26.3 (5.8)	51.4
Quintile 3	19.5	27.5 (5.3)	68.2	20.1	26.7 (6.3)	54.2
Quintile 2	18.9	27.5 (5.2)	68.3	19.3	27.0 (6.4)	54.9
Quintile 1 (most disadvantage)	17.5	27.4 (5.7)	64.9	17.2	28.0 (7.2)	60.8

Countries of birth of respondents comprising >5 % of region sample

Oceania: New Zealand, Fiji, Papua New Guinea

North-West Europe: United Kingdom, Netherlands, Germany

Southern & Eastern Europe: Italy, Poland, Croatia, Federal Republic of Yugoslavia, Romania, Former Yugoslav Republic of Macedonia

North Africa & Middle East: Lebanon, Egypt, Turkey, Iraq, Iran

South-East Asia: Philippines, Vietnam, Malaysia, Indonesia

North-East Asia: China, Hong Kong, Japan, Taiwan

Southern & Central Asia: India, Sri Lanka, Nepal, Bangladesh, Pakistan

Americas: USA, Canada, Chile, Colombia

Sub-Saharan Africa: South Africa, Mauritius, Zimbabwe

*Abbreviations: BMI* body mass index, *Owt* overweight, *SD* standard deviation

#### Controls

Six demographic and socioeconomic variables were included in the models as controls to address potential sources of confounding as identified in the literature [5, 7, 9, 28]. Variables were categorised as shown in Table 1 and included age (date of birth), highest educational qualification, occupation and annual household income, with data collected through interviewer-administered questionnaires [41]. Neighbourhood disadvantage was derived by the data provider from a ranking based on the Australian Bureau of Statistics’ methods of compiling a range of indicators of socio-economic disadvantage into a single ‘SEIFA’ (Socio-Economic Indexes for Areas) index [47]. For this study the SEIFA 2011 Decile of Index of Relative Socio-economic Disadvantage (IRSD) was used to calculate quintiles of neighbourhood disadvantage. Area remoteness was derived by the data-provider and based on the Australian Standard Geographical Classification (ASGC) [48]. Remoteness was included as a control variable as it is an element of disadvantage that has been linked to obesity in Australia [49, 50].

### Analysis

The analysis comprised two stages: the first examined the relationship between ethnicity (country of birth) and bodyweight; and the second stage examined acculturation (length of stay and age at arrival) and bodyweight.

#### Ethnicity and bodyweight

Those who were aged < 18 years or were pregnant in the last year were removed from the sample (*n* = 1 751) as out of scope for this study. Those who had no self-completed questionnaire, had missing or implausible BMI data or missing data for the predictor variables (*n* = 2 814) were also excluded from the analysis: of these, predictors for non-inclusion were younger age (18–24 and 25–34 years) (*p* < 0.001) and those born in Oceania (*p* = 0.041), Southern & Eastern Europe (*p* < 0.001), North Africa & Middle East (*p* < 0.001) and South East Asia (*p* = 0.001). The final analytic sample included 13 047 adults (6 216 men and 6 831 women). We examined the relationship between ethnicity and bodyweight using a staged modelling approach and stratifying by gender. The staged approach included a base model (model 1) with only country of birth and subsequently adjusted for age (model 2) and adjusted for socioeconomic variables and area remoteness (model 3). The reference group was Australian born. Multilevel linear and logistic regression techniques were selected due to the multi-level structure of the data and to account for clustering at the individual, household, neighbourhood and area levels. Regression analyses were used to examine associations between the outcome variables (BMI, overweight/obesity) and the predictor variables. We tested for an interaction between ethnicity and sex on BMI. The parameters for the multilevel logistic models were estimated using Markov Chain Monte Carlo (MCMC) simulation [51]. Results are presented as odds ratios and their 95 % credible intervals (CrI). All models were run with sufficient iterations to meet the minimum estimation requirements. The statistical analyses were performed using STATA 12 and MLwiN [52].

#### Acculturation and bodyweight

For the second stage of the analysis, we took the analytic sample from the first stage and excluded those born in Australia, resulting in a sample of 2 997 foreign-born adults (1 457 men and 1 540 women). The modelling approach was the same as previous, with the addition of a further model (model 4) adjusting for country of birth. The hypothesised least acculturated group was used as the reference category: length of residence < 5 years, and age at arrival *≥* 25 years. Linear and logistic regression analyses were used to model BMI and odds overweight/obesity as per previous.

## Results

### Ethnicity and bodyweight

Table 1 describes the summary characteristics of the analytic sample (*n* = 13 047). The majority of the sample were Australian born (76.5 and 77.4 % for men and women), which is broadly reflective of their proportion in the Australian population. The majority were middle aged (35–64 years) and lived in either major cities or inner regional centres. Nearly half of the women (48.2 %) had low educational attainment. The highest proportions of overweight and obesity were seen in men and women born in Southern and Eastern Europe (74.7 and 64.5 % respectively). Male and female immigrants from North Africa/Middle East had the highest mean BMIs of 28.8 kg m^−2^ (SD 8.5) and 28.0 kg m^−2^ (SD 6.2) respectively. Significant interaction effects for gender and ethnicity on BMI (*p* < 0.001), and on percent overweight/obese (*p* = 0.004) were found, therefore analyses were stratified by gender.

Among men, after adjustment for age, area remoteness, education, occupation, household income and neighbourhood disadvantage, BMI was significantly higher for immigrants from North Africa/Middle East (β = 1.42, 95 % confidence interval (CI) = 0.19, 2.64) and Oceania (β = 0.84, CI = 0.16, 1.52), compared with Australian born (Table 2). Men from North West Europe (β = −0.47, CI = −0.89, −0.05), North East Asia (β = −1.48, CI = −2.63, −0.34) and Southern and Central Asia (β = −1.24, CI = −2.17, −0.32) had significantly lower BMIs relative to Australian born. In the fully adjusted models, the odds of being overweight/obese were significantly less among Asian ethnic groups.

**Table 2:** Country of birth by BMI and Odds Overweight/Obesity, men and women

	BMI						Odds Overweight/Obesity					
	Model 1^a^		Model 2^b^		Model 3^c^		Model 1^a^		Model 2^b^		Model 3^c^	
	Coeff	95 % CI	Coeff	95 % CI	Coeff	95 % CI	OR	95 % CrI	OR	95 % CrI	OR	95 % CrI
Men												
Australian born	Reference		Reference		Reference		Reference		Reference		Reference	
Oceania (excluding Australia)	**1.31**	(0.62, 2.01)	**0.89**	(0.21, 1.58)	**0.84**	(0.16, 1.52)	1.35	(1.00, 1.87)	1.14	(0.83, 1.59)	1.12	(0.81, 1.57)
North-West Europe	−0.10	(−0.53, 0.32)	**−0.52**	(−0.94, −0.10)	**−0.47**	(−0.89, −0.05)	1.10	(0.92, 1.33)	**0.87**	(0.72, 0.88)	0.88	(0.73, 1.08)
Southern & Eastern Europe	0.67	(−0.10, 1.45)	0.23	(−0.53, 1.00)	0.24	(−0.52, 1.01)	**1.59**	(1.11, 2.31)	1.29	(0.89, 1.91)	1.33	(0.91, 1.97)
N. Africa & The Middle East	**1.69**	(0.43, 2.94)	**1.25**	(0.03, 2.47)	**1.42**	(0.19, 2.64)	1.20	(0.70, 2.11)	0.99	(0.56, 1.81)	1.12	(0.63, 2.08)
South-East Asia	−0.73	(−1.70, 0.23)	**−1.03**	(−1.97, −0.09)	−0.90	(−1.84, 0.05)	**0.61**	(0.41, 0.90)	**0.53**	(0.35, 0.79)	**0.57**	(0.37, 0.86)
North-East Asia	**−1.77**	(−2.94, −0.59)	**−1.75**	(−2.89, −0.60)	**−1.48**	(−2.63, −0.34)	**0.35**	(0.21, 0.57)	**0.33**	(0.20, 0.55)	**0.36**	(0.21, 0.61)
Southern & Central Asia	**−1.25**	(−2.19, −0.32)	**−1.44**	(−2.36, −0.52)	**−1.24**	(−2.17, −0.32)	**0.58**	(0.39, 0.84)	**0.53**	(0.36, 0.78)	**0.59**	(0.39, 0.88)
Americas	**1.18**	(0.02, 2.35)	0.69	(−0.45, 1.83)	0.97	(−0.16, 2.11)	1.37	(0.81, 2.37)	1.11	(0.65, 1.97)	1.23	(0.72, 2.16)
Sub-Saharan Africa	−0.94	(−2.09, 0.21)	−0.95	(−2.07, 0.18)	−0.77	(−1.89, 0.35)	0.78	(0.48, 1.28)	0.78	(0.47, 1.30)	0.81	(0.49, 1.37)
Women												
Australian born	Reference		Reference		Reference		Reference		Reference		Reference	
Oceania (excluding Australia)	0.27	(−0.67, 1.22)	−0.19	(−1.11, 0.73)	−0.22	(−1.13, 0.70)	1.22	(0.90, 1.68)	1.05	(0.77, 1.45)	1.05	(0.76, 1.45)
North-West Europe	−0.25	(−0.79, 0.28)	**−0.91**	(−1.44, −0.38)	**−0.80**	(−1.33, −0.28)	0.95	(0.79, 1.13)	**0.72**	(0.60, 0.87)	**0.75**	(0.62, 0.90)
Southern & Eastern Europe	0.69	(−0.26, 1.64)	−0.07	(−1.00, 0.86)	−0.12	(−1.05, 0.80)	**1.59**	(1.15, 2.21)	1.21	(0.87, 1.70)	1.20	(0.87, 1.69)
N. Africa & The Middle East	1.47	(−0.37, 3.31)	0.89	(−0.91, 2.69)	0.73	(−1.06, 2.52)	1.52	(0.81, 2.92)	1.36	(0.72, 2.60)	1.31	(0.69, 2.55)
South-East Asia	**−1.96**	(−2.89, −1.04)	**−2.35**	(−3.27, −1.45)	**−2.34**	(−3.25, −1.43)	**0.45**	(0.33, 1.60)	**0.41**	(0.29, 0.56)	**0.41**	(0.30, 0.57)
North-East Asia	**−4.92**	(−6.08, −3.76)	**−4.99**	(−6.13, −3.85)	**−4.93**	(−6.06, −3.79)	**0.11**	(0.06, 0.19)	**0.11**	(0.06, 0.18)	**0.11**	(0.06, 0.19)
Southern & Central Asia	**−1.36**	(−2.57, −0.15)	**−1.54**	(−2.73, −0.36)	**−1.60**	(−2.77, −0.42)	0.67	(0.44, 1.01)	**0.63**	(0.41, 0.96)	**0.64**	(0.42, 0.97)
Americas	**−1.27**	(−2.52, −0.02)	**−1.81**	(−3.03, −0.59)	**−1.67**	(−2.88, −0.45)	0.73	(0.48, 1.12)	**0.62**	(0.41, 0.95)	0.66	(0.43, 1.00)
Sub-Saharan Africa	**−2.34**	(−3.82, −0.86)	**−2.54**	(−3.99, −1.09)	**−2.37**	(−3.81, −0.93)	**0.45**	(0.27, 0.75)	**0.42**	(0.24, 0.71)	**0.44**	(0.26, 0.74)

*Abbreviations: BMI* body mass index, *CI* confidence interval, *OR* odds ratio, *CrI* credible interval

^a^Base model, no adjustment. ^b^Adjusted for age. ^c^Adjusted for age, area remoteness, education, occupation, household income, neighbourhood disadvantage. Bold *p* < 0.05

Among women, six of the nine ethnic immigrant groups had significantly lower BMIs compared with the Australian-born reference group. The results for odds overweight/obesity showed similar patterns. Immigrants from North East Asia had the largest (lower) BMI difference compared with Australian-born (β = −4.93, CI = −6.06, −3.79).

### Acculturation and bodyweight

Table 3 describes the summary characteristics of the foreign born sample (*n* = 2 997), which includes small proportions in the youngest age category (18–24 years) and the majority living in major cities. Over 40 % of women were in the lowest category for individual measures of socioeconomic position (education and occupation). Over 75 % of the foreign born sample had lived in Australia for >15 years and the majority arrived as adults (*≥*25 years). Those who had resided in Australia for 15 or more years, had the highest BMIs in both males and females (27.3 (5.1) and 26.4 (5.8) respectively). Men who arrived during adolescence had a relatively high BMI of 28.0 (6.2) and both men and women who arrived as children (0–11 years), also had high BMIs compared with the other categories. The %overweight/obese descriptive results for the acculturation variables showed the same patterns as for mean BMI.

**Table 3:** Socio-demographic and bodyweight characteristics of men and women in the acculturation and bodyweight sample (*n* = 2 997)

	Men (FB Only) (*n* = 1 457, 48.6 %) 27.1(5.2) mean BMI(SD) 64.9%Owt/Obese			Women (FB Only) (*n* = 1 540, 51.4 %) 25.9 (6.0) mean BMI(SD) 47.8%Owt/Obese		
	%	Mean BMI (SD)	%Owt/ Obese	%	Mean BMI (SD)	%Owt/ Obese
Length of residence in Australia						
< 5 years	6.7	25.3 (4.1)	49.0	9.1	23.2 (4.2)	25.7
5–9 years	6.2	25.8 (4.6)	57.1	5.8	24.9 (9.0)	31.5
10–14 years	7.8	26.7 (6.2)	56.6	9.2	24.7 (5.2)	41.1
≥ 15 years	79.3	27.3 (5.1)	67.6	76.0	26.4 (5.8)	52.4
Age at Arrival						
≥ 25 years (arrived as adult)	44.9	26.7 (5.1)	62.6	45.1	25.5 (5.8)	46.2
18–24 years (arrived as young adult)	18.8	26.7 (4.6)	62.6	19.7	25.2 (5.1)	44.2
12–17 years (arrived as an adolescent)	8.0	28.0 (6.2)	70.1	9.0	26.2 (5.5)	50.0
0–11 years (arrived as young child)	28.3	27.5 (5.3)	68.5	26.2	27.0 (6.8)	52.5
Age						
18–24 years	5.4	24.9 (4.2)	40.5	4.7	22.4 (4.1)	23.3
25–34 years	11.9	25.9 (4.6)	50.0	12.3	23.9 (5.7)	28.6
35–44 years	15.0	27.2 (4.2)	65.3	16.9	25.6 (5.9)	43.8
45–54 years	22.5	27.3 (5.0)	68.9	21.8	25.7 (5.8)	46.7
55–64 years	20.6	28.0 (5.5)	73.7	21.9	27.0 (6.7)	54.3
65–74 years	15.5	27.5 (5.6)	69.0	13.8	27.2 (5.4)	60.6
> 75 years	9.0	26.2 (4.6)	61.1	8.6	26.7 (4.9)	62.1
Remoteness						
Major City	76.9	27.0 (5.1)	64.5	77.9	25.8 (6.0)	46.7
Inner Regional Australia	15.8	27.3 (5.7)	64.1	13.9	26.1 (5.2)	51.4
Outer Regional Australia	5.9	27.2 (4.9)	68.6	6.8	27.0 (6.7)	52.4
Remote and Very Remote Australia	1.3	27.9 (4.0)	78.9	1.4	24.9 (5.1)	48.6
Highest attained education level						
Bachelor +	32.3	26.7 (5.3)	61.1	33.6	24.6 (5.1)	38.8
Diploma	11.0	26.7 (4.8)	60.6	10.7	25.1 (6.2)	41.2
Certificate (trade/business)	24.9	27.3 (4.4)	70.8	13.4	26.7 (6.4)	53.4
School - Year 12 and below	31.8	27.4 (5.6)	65.5	42.3	26.9 (6.2)	54.8
Occupation						
Managers and professionals	29.2	26.8 (4.7)	65.0	22.5	25.0 (4.9)	40.9
White Collar	12.8	26.9 (5.5)	61.5	23.6	25.4 (6.1)	41.8
Blue Collar	25.0	27.3 (4.8)	65.1	7.3	26.0 (6.5)	42.9
Unemp/Not in Labour Force	32.9	27.2 (5.7)	65.8	46.6	26.5 (6.2)	54.9
Household Income						
> $130,000 k per annum	18.3	26.8 (4.4)	61.3	15.7	24.7 (5.4)	38.4
$72,800–$129,999	35.4	27.4 (5.2)	68.6	33.1	25.6 (5.5)	46.9
$52,000–$72,799	16.3	26.2 (4.5)	58.6	15.9	25.8 (6.3)	43.3
$41,600–$51,999	7.6	27.1 (5.7)	63.1	7.5	25.9 (5.4)	44.8
$26,000–$41,599	14.1	27.6 (6.1)	68.0	14.3	26.8 (6.7)	55.4
$0–$25,999	8.3	27.0 (5.4)	65.3	13.4	27.0 (6.4)	59.9
Neighbourhood Disadvantage						
Quintile 5 (least disadvantage)	24.8	26.5 (3.7)	62.7	23.6	24.3 (4.7)	36.1
Quintile 4	20.4	26.7 (4.8)	63.8	21.3	25.8 (5.4)	48.2
Quintile 3	16.7	28.0 (6.3)	68.7	18.1	26.1 (6.5)	48.2
Quintile 2	20.0	27.5 (5.7)	70.8	19.5	25.9 (5.9)	48.2
Quintile 1 (most disadvantage)	18.0	26.8 (5.4)	58.9	17.5	27.8 (7.0)	62.2

*Abbreviations: BMI* body mass index, *FB* foreign born, *Owt* overweight, *SD* standard deviation

Length of residence in Australia: After adjustment for age, area remoteness, education, occupation, household income, neighbourhood disadvantage and nativity, male immigrants who had lived in Australia for ≥ 15 years had significantly higher BMIs (β = 1.27, CI = 0.10, 2.44) compared with their counterparts residing in Australia less than 5 years (Table 4). Among female immigrants, as length of residence increased, so too did odds of overweight/obesity, however the relationship reached significance only for those living in Australia for ≥ 15 years (odds ratio (OR) = 1.59, 95 % credible interval (CrI) = 1.04, 2.46). Odds ratios were not significant for men and BMI results were not significant for women.

**Table 4:** Length of residence in Australia by BMI and Odds Overweight/Obesity, men and women

	BMI								Odds Overweight/Obesity							
	Model 1^a^		Model 2^b^		Model 3^c^		Model 4^d^		Model 1^a^		Model 2^b^		Model 3^c^		Model 4^d^	
	Coeff	95 % CI	Coeff	95 % CI	Coeff	95 % CI	Coeff	95 % CI	OR	95 % CrI	OR	95 % CrI	OR	95 % CrI	OR	95 % CrI
Men (FB only)																
< 5 years	Reference		Reference		Reference		Reference		Reference		Reference		Reference		Reference	
5–9 years	0.63	(−0.84, 2.10)	0.63	(−0.82, 2.08)	0.71	(−0.73, 2.16)	0.61	(−0.82, 2.05)	1.43	(0.87, 2.37)	1.41	(0.84, 2.34)	1.56	(0.90, 2.73)	1.56	(0.91, 2.70)
10–14 years	**1.47**	(0.05, 2.88)	1.18	(−0.25, 2.61)	1.31	(−0.11, 2.74)	0.95	(−0.47, 2.37)	1.37	(0.85, 2.22)	1.06	(0.65, 1.73)	1.15	(0.67, 2.0)	1.03	(0.61, 1.78)
≥ 15 years	**2.05**	(0.96, 3.14)	**1.45**	(0.28, 2.61)	**1.54**	(0.37, 2.71)	**1.27**	(0.10, 2.44)	**2.20**	(1.53, 3.16)	1.43	(0.95, 2.16)	1.54	(0.98, 2.44)	1.33	(0.86, 2.08)
Women (FB only)																
< 5 years	Reference		Reference		Reference		Reference		Reference		Reference		Reference		Reference	
5–9 years	1.53	(−0.01, 3.07)	0.97	(−0.57, 2.52)	0.86	(−0.67, 2.40)	1.13	(−0.38, 2.64)	1.28	(0.75, 2.15)	1.09	(0.64, 1.83)	1.06	(0.61, 1.82)	1.17	(0.66, 2.06)
10–14 years	1.24	(−0.14, 2.64)	0.56	(−0.85, 1.97)	0.34	(−1.06, 1.73)	0.19	(−1.17, 1.56)	**1.97**	(1.24, 3.11)	1.54	(0.96, 2.42)	1.47	(0.91, 2.37)	1.43	(0.87, 0.42)
≥ 15 years	**2.86**	(1.81, 3.91)	**1.32**	(0.13, 2.50)	1.13	(−0.06, 2.31)	0.77	(−0.40, 1.93)	**3.17**	(2.19, 4.60)	**1.79**	(1.21, 2.61)	**1.75**	(1.17, 2.63)	**1.59**	(1.04, 2.46)

*Abbreviations: BMI* body mass index, *Coeff* coefficient, *CI* confidence interval, *CrI* credible interval, *FB* foreign born, *OR* odds ratio

^a^Base model, no adjustment. ^b^Adjusted for age. ^c^Adjusted for age, area remoteness, education, occupation, household income, neighbourhood disadvantage. ^d^Adjusted for age, area remoteness, education, occupation, household income, neighbourhood disadvantage, country of birth. Bold *p* < 0.05

#### Age at arrival

After full adjustment, male immigrants who arrived as a young child or an adolescent had significantly higher BMIs (β = 1.27, CI = 0.59, 1.95 and β = 2.01, CI = 1.03, 3.06 respectively) and odds overweight/obesity (OR = 1.65, CrI = 1.26, 2.16 and OR = 2.09, CrI = 1.38, 3.18 respectively) compared with immigrants who arrived as adults (Table 5). Among women, immigrants who arrived as a young child also had significantly higher BMIs (β = 1.67, CI = 0.92, 2.42) and odds overweight/obesity (OR = 1.45, CrI = 1.12, 1.86) compared with those who arrived as adults.

**Table 5:** Age at arrival in Australia by BMI and Odds Overweight/Obesity, men and women

	BMI								Odds Overweight/Obesity							
	Model 1^a^		Model 2^b^		Model 3^c^		Model 4^d^		Model 1^a^		Model 2^b^		Model 3^c^		Model 4^d^	
	Coeff	95 % CI	Coeff	95 % CI	Coeff	95 % CI	Coeff	95 % CI	OR	95 % CrI	OR	95 % CrI	OR	95 % CrI	OR	95 % CrI
Men (FB only)																
≥ 25 years (arr. as adult)	Reference		Reference		Reference		Reference		Reference		Reference		Reference		Reference	
18–24 years (arr. as young adult)	−0.08	(−0.81, 0.64)	0.23	(−0.49, 0.95)	0.07	(−0.66, 0.79)	−0.08	(−0.80, 0.64)	1.01	(0.78, 1.30)	1.23	(0.90, 1.71)	1.11	(0.84, 1.47)	1.07	(0.81, 1.42)
12–17 years (arr. as adolescent)	**1.20**	(0.20, 2.21)	**1.86**	(0.85, 2.88)	**1.92**	(0.91, 2.94)	**2.01**	(1.03, 3.06)	1.42	(0.98, 2.08)	**2.31**	(1.43, 3.88)	**2.09**	(1.38, 3.18)	**2.09**	(1.38, 3.18)
0–11 years (arr. as young child)	**0.63**	(0.01, 1.26)	**1.23**	(0.58, 1.89)	**1.19**	(0.53, 1.86)	**1.27**	(0.59, 1.95)	**1.28**	(1.02, 1.62)	**1.92**	(1.42, 2.66)	**1.78**	(1.37, 2.33)	**1.65**	(1.26, 2.16)
Women (FB only)																
≥ 25 years (arr. as adult)	Reference		Reference		Reference		Reference		Reference		Reference		Reference		Reference	
18–24 years (arr. as young adult)	−0.38	(−1.17, 0.40)	0.36	(−0.42, 1.14)	0.42	(−0.36, 1.20)	0.17	(−0.61, 0.94)	0.90	(0.71, 1.15)	1.15	(0.90, 1.48)	1.18	(0.91, 1.53)	1.05	(0.81, 1.37)
12–17 years (arr. as adolescent)	0.29	(−0.78, 1.35)	0.57	(−0.47, 1.61)	0.53	(−0.50, 1.56)	0.30	(−0.73, 1.32)	1.08	(0.78, 1.50)	1.22	(0.88, 1.72)	1.22	(0.86, 1.72)	1.08	(0.76, 1.52)
0–11 years (arr. as young child)	**1.25**	(0.53, 1.96)	**2.17**	(1.45, 2.90)	**2.16**	(1.44, 2.89)	**1.67**	(0.92, 2.42)	1.24	(0.99, 1.55)	**1.73**	(1.38, 2.19)	**1.77**	(1.39, 2.27)	**1.45**	(1.12, 1.86)

*Abbreviations: Arr* arrived, *BMI* body mass index, *Coeff* coefficient, *CI* confidence interval, *CrI* credible interval, *FB* foreign born, *OR* odds ratio

^a^Base model, no adjustment. ^b^Adjusted for age. ^c^Adjusted for age, area remoteness, education, occupation, household income, neighbourhood disadvantage. ^d^Adjusted for age, area remoteness, education, occupation, household income, neighbourhood disadvantage, country of birth. Bold *p* < 0.05

## Discussion

This study revealed gender-specific ethnic differences in bodyweight in a national sample of Australian adults. Adjustment for socioeconomic factors had minimal and variable impact on regression coefficients and odds ratios, suggesting that these constructs do not explain ethnic differences in bodyweight in Australia. Two ethnic groups had significantly higher BMI compared with Australian born - male immigrants born in North Africa/Middle East, and Oceania. This contrasts to results from single-State Australian studies, which identified immigrants born in Southern European countries as having significantly higher BMIs compared with Australian born after full adjustment [23, 24]. Published results from these earlier studies were not stratified by gender and comparisons are difficult due to methodological constraints (as described in the introduction). There is a paucity of international studies focused on immigrants from Oceania and North Africa/Middle Eastern regions. International prevalence data has shown in excess of 50 % obesity rates in countries of these regions [1], however it cannot be assumed that nationals living in their own countries have the same characteristics as those who immigrate, underscoring the importance of further research on ethnic differences in bodyweight among existing and emerging immigrant cohorts.

Findings from this study of lower BMI among male and female Asian immigrants compared with Australian born are generally consistent with State-based research [23, 24]. It remains important however, to include Asian immigrants in obesity monitoring and prevention efforts, as using Asian BMI cut-offs for overweight/obesity has revealed higher levels of health risks, [25] and generational studies from multiple countries have shown a rapid upward assimilation of Asian immigrants’ BMI to the host country’s BMI over the course of one generation [14, 24, 53].

The acculturation results demonstrated that Australian immigrants are no exception to international evidence of immigrants having lower BMI on arrival and increasing BMI with longer durations of residence. A number of contributing factors have been postulated to explain these phenomena. These include, strict immigrant health entry requirements [54]; protective biological, behavioural and sociocultural factors [54, 55]; and immigrant self-selection, that is, only those who are healthy, educated and have the financial means to migrate, do so [56]. Most studies from developed countries found that women are particularly susceptible to increasing BMI with longer duration of residence [28, 29, 57, 58], although others have found the opposite [28]. The modelling in this study showed that the relationship between duration of residence and BMI was significant only for men living in Australia for ≥ 15 years and that among women, socioeconomic factors and nativity explained the increased BMI with increased length of residence. The odds of overweight/obesity remained significantly higher after full adjustment among female immigrants residing in Australia ≥ 15 years, which suggests that conclusions on gender acculturation differences may vary depending on the measures used to assess adiposity.

Age at arrival results from this study supported the length of residence results which in turn is consistent with findings that arrival < 20 years of age (compared with arrival at later ages), placed immigrants at higher risk of overweight/obesity [40]. This study is unique in showing that men who arrived as adolescents are at particularly high risk in terms of their adult BMI and likelihood of overweight/obesity, suggesting an important area for policy attention.

### Strengths, limitations and areas for further research

This study had a number of limitations. Country of birth is the most commonly used indicator of ethnicity in Australian datasets and other, more sensitive measures such as self-identified ethnicity [59] are not routinely gathered. Country of birth may be only one of several factors which influence a person’s ethnicity [60] and in this study, aggregating countries into regions may mask important heterogeneity both within countries and within regions. The self-completed questionnaire was only available in English and analysis of reasons for exclusion, revealed that birth in non-English speaking regions may be an important predictor of questionnaire non-return and may have introduced selection bias into the sample. Self-reported BMI is known to be subject to error [61] and further research is needed to confirm the presence and direction of weight-reporting biases among adults in different ethnic groups [28, 62]. In this study, as we were comparing ethnic differences in overweight and obesity relative to Australian born, the WHO standard overweight and obesity cut-off points were used. This may underestimate overweight and obesity amongst some Asian ethnic groups [63].

Acknowledging the constraints of this paper, the complexity of ethnicity as a construct and the under-representation of ethnic minority groups in health research, we echo the calls of other researchers [54, 59] for increased population-level research on migrant health trends and the inclusion of a greater range of ethnicity variables and appropriate data collection techniques to enable this to occur. Findings from this study, along with research from other developed countries, suggest that the complex and intertwining nature of ethnicity, acculturation, gender and socioeconomic status requires further context specific research. In particular longitudinal studies will build on our findings and reveal trends which take into account cohort effects and secular and age-related increases in obesity [64].

## Conclusions

This paper was the first study of its kind to examine ethnic differences in BMI and overweight/obesity and the influence of acculturation on the bodyweight of immigrants in a national sample of Australian adults. Our findings emphasise the importance of targeted and tailored obesity prevention intervention aimed at ethnic groups at high risk of overweight and obesity. In the Australian context this includes male immigrants from North Africa/Middle East and Oceania regions. Our findings also highlight the need for public health policy directed at immigrants in the early years post-arrival and to those who arrive as young children or adolescents, in order to combat acculturation-related weight gain. The study adds to the international literature by demonstrating the pervasiveness of ethnic differences in immigrant bodyweight and the consistency and speed of immigrant acculturation to a country’s unhealthy weight profile in the face of obesogenic environments present in developed countries.

## Abbreviations

BMI: Body mass index
CrI: Credible interval
HILDA: Household, income and labour dynamics in Australia
MCMC: Markov chain Monte Carlo
OR: Odds ratio
WHO: World Health Organisation
